# Impact of organic and integrated production systems on yield and seed quality of rainfed crops and on soil properties

**DOI:** 10.3389/fnut.2023.1127970

**Published:** 2023-05-10

**Authors:** Kodigal A. Gopinath, Govindarajan Venkatesh, Mallappa Manjunath, Mitnala Jayalakshmi, Telaprolu Venkata Prasad, Bollam Rajkumar, Visha Kumari Venugopalan, Bellapukonda Murali Krishna Raju, Mathyam Prabhakar, Gajjala Ravindra Chary, Vinod Kumar Singh

**Affiliations:** ^1^ICAR-Central Research Institute for Dryland Agriculture (CRIDA), Hyderabad, India; ^2^Regional Agricultural Research Station (RARS), Acharya NG Ranga Agricultural University, Nandyal, India

**Keywords:** organic production system, pigeonpea, greengram, sunflower, soil health, rainfed areas

## Abstract

Mineral and vitamin deficiencies together affect a greater number of human populations in the world than does protein malnutrition. Organic farming is reported to improve nutritional quality of food grains while also improving soil health. However, sufficient scientific information on several aspects of organic farming based on long-term studies is lacking particularly under rainfed conditions of India. The purpose of this study was to assess the long-term impact of organic and integrated production systems on crops yield and quality, economic returns and soil properties. The study was conducted with three crops, sunflower (*Helianthus annuus* L.), pigeonpea (*Cajanus cajan* L.), and greengram [*Vigna radiata* (L.) Wilczek] under three different production systems, control (use of chemical inputs alone), organic and integrated. The results of the 10-year study revealed that, the average production of integrated system was on par with organic management and recorded significantly higher pigeonpea equivalent yield (PEY) (827 kg ha^−1^) compared to control (chemical inputs) (748 kg ha^−1^). In general, the yield gap between organic and integrated production systems declined from fourth year for greengram and eighth year for sunflower, during the 10-year experimental period whereas the pigeonpea yield was similar under both production systems from first year. Plots under organic management had significantly lower bulk density (1.18 mg m^−3^), higher water holding capacity (38.72%) and porosity (53.79%) compared to integrated production system and control (chemical inputs). The soil organic C (SOC) content in the plots under organic production system was 32.6% more than the initial organic carbon of the soil (0.43%), with higher soil N (205.2 kg ha^−1^). Plots under integrated production system, however, had higher soil P (26.5 kg ha^−1^) compared with other treatments. The dehydrogenase activity (5.86 μg TPF g^−1^ soil h^−1^) and microbial biomass carbon (317.3 μg g^−1^ soil) content was higher in the plots under organic production system than under other systems. Organically produced pigeonpea and greengram seeds had similar protein content with that of integrated system, and higher K and micronutrient (Fe, Zn, Cu, and Mn) contents than other treatments. The results show the potential of organic production system in improving crop yields, soil properties and produce quality in semiarid rainfed areas.

## Introduction

Several countries have started promoting organic farming as an alternative to high-input agriculture (conventional farming). Organic farming is one of the fastest growing sectors of agricultural production. As per the latest FiBL survey, 74.9 million ha were under organic agricultural management worldwide (1). Organic farming is not new to India as this nature friendly farming practice is done in the country from ancient times (2). Recently, promotion of organic farming is one of the priority areas of Government of India to improve agricultural productivity while reducing the use of external inputs; The Government is promoting adoption of organic farming in India through various schemes such as Paramparagat Krishi Vikas Yojana (PKVY) and Mission Organic Value Chain Development for North East Region (MOVCDNER). Last decade witnessed a huge jump under the area of certified organic farming in India. Presently, India has about 2.7 million ha under certified organic farming with the highest number of organic producers (1.6 million) in the world (1).

The productivity of rainfed agriculture which constitutes about 51% of the cultivated area in India is constrained by the aberrant monsoon, low and unstable yield, small farm size, degraded soil and resource poor farmers. Smallholders in rainfed regions may have the chance to increase their output through organic farming without relying on outside resources like capital or inputs, and they may also be able to sell their food for higher prices. Numerous studies have compared the output, impact on the environment, and financial returns of organic and conventional farming. In general, some loss in crop yields is observed after discarding synthetic inputs and converting the operations from the conventional systems to organic production (3, 4), while others have reported that organic systems can be as productive as conventional ones (5, 6). By adding organic manures, the soil’s available nutrients usually benefit in the form of increased yields (7, 8). However, literature on performance of rainfed organic production systems is scanty. Though organic farming systems are low-impact and low-yielding than conventional or integrated management systems, they are reported to be more resilient and offer nutrient-dense quality food (9, 10). Further, the reduction in crop yields from organic systems can very well be compensated by the higher economic returns fetching from the price premium (11, 12).

On the other hand, organic amendments like farmyard manure (FYM), vermicompost and green manures lowers bulk density, improves porosity and infiltration rates, reduces surface runoff, increase water-holding capacity thus improving soil physical properties (13–16). Furthermore, numerous studies demonstrate that soil fertility is increased over time by organic farming (14, 17–19). In comparison to conventionally maintained systems, these organic systems also result in superior soil quality and greater soil biological activity (19, 20). Unlike chemical fertilizers, organic amendments are characterized with their slower nutrient release pattern coupled with higher residual effect on the subsequent crops (21, 22). Judicious application of organic amendments improve the crop productivity in addition to maintaining the sustainability of the system (23, 24) because of the organic manure being the basic source of organic matter in soil. In fact, one of the greatest challenges in the present world is to feed the ever-increasing population, still maintaining soil health along with environmental quality (25).

Legume and oilseed crops are the most relevant crop type in global food security, and as such, the move toward resilient and more sustainable cropping systems by reducing the chemical input is a major challenge. Sunflower (*Helianthus annuus* L.), greengram [*Vigna radiata* (L.) Wilczek] and pigeonpea [*Cajanus cajan* (L.) Millsp.] are well suited for the rainfed regions of semi-arid tropics and are widely grown in this region. Further, no attempts were made so far to assess the impact of different production systems on performance of these crops and on different soil properties. Hence, we carried out a study to assess the impact of organic and conventional production systems on performance of sunflower, greengram and pigeonpea, crop quality and soil properties in semiarid rainfed conditions. Here, the first hypothesis we tested was that organic production system would improve crops yield and quality compared to that of conventional production systems due to improvement of soil properties. The second hypothesis tested was that crops respond differently to different production systems.

## Materials and methods

### Study area

**Figure 1:**
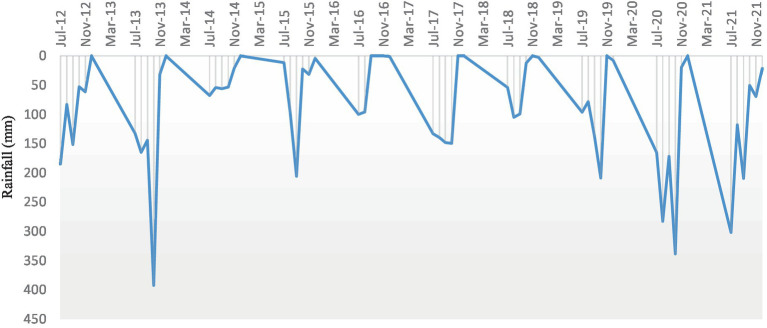
Monthly total rainfall (mm) during the cropping period (July–December) during 2012–2021.

**Figure 2:**
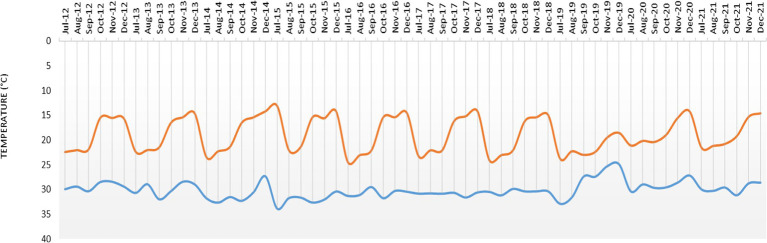
Mean monthly maximum and minimum temperature during the crop period (July–December) during 2012–2021.

The study location is situated in India’s 7.2 agro-ecological subregion, and the growing season lasts between 120 and 150 days. The region has a semi-arid (dry) climate with three distinct seasons: the summer (March to May), the rainy season (*kharif*), which lasts from June to September, and the winter (*rabi*) (October to February). At the Gungal Research farm of the ICAR-Central Research Institute for Dryland Agriculture (17°40′ 40.4″ N latitude and 78°39′, 55.7″ E longitude and at a mean sea level of 626 m), Hyderabad, Telangana, India, the field experiment was carried out for 10 years between 2012 and 2021.The farm represents a semi-arid tropical region with a mean annual temperature of 25.7°C and rainfall of 746 mm. The monthly rainfall during the crop season (July–December) during the study period (2012–2021) and the monthly maximum and minimum temperature prevailed during the period are given in Figures 1, 2. Soil of the experimental site is sandy loam; slightly acidic in reaction (pH 6.51), EC was in normal range (0.05–0.07 dS m^−1^), low in organic carbon (0.43%), available N (229.1 kg ha^−1^), high in available P (24.7 kg ha^−1^), and medium in available K (218.1 kg ha^−1^) (3).

### Treatments

Three production systems *viz.* organic, integrated and control (chemical inputs), and three field crops *viz.* sunflower (*Helianthus annuus* L.), pigeonpea [*Cajanus cajan* (L.) Millsp.], and greengram [*Vigna radiata* (L) Wilczek] were studied in this experiment every year. The experiment was laid out in strip plot design. All the treatments were replicated thrice in a plot size of 12 m × 4 m. The package of practices in each crop are presented in Table 1. The FYM was sourced from the same place every year and the average composition of 0.5% N, 0.25% P, 0.4% K, 27.9 ppm Cu, 228.7 ppm Mn, 452 ppm Fe, and 143.1 ppm Zn (3).

**Table 1:** Variety, seed rate and planting geometry used for each crop.

Crop	Variety	Seed rate (kg ha^−1^)	Planting geometry (cm)
Sunflower	DRSH-1	6	60 × 30
Greengram	WGG-37	15	30 × 15
Pigeonpea	PRG-158	15	90 × 20

The FYM was treated with *Trichoderma viridae* at 2.5 kg ha^−1^, as a prophylactic measure against soil borne diseases as explained by Gopinath et al. (26). The details of application of nutrients in different treatments are given in Tables 2, 3. Under integrated method, one fourth of the N was applied through FYM. The remaining N and total P and K was applied through mineral fertilizers. In sunflower crop, N was applied in basal and two other splits (30 and 60 DAS). However, in pigeonpea and greengram the nutrients were applied basal.

**Table 2:** Application rates of farmyard manure and mineral fertilizers in different treatments.

Treatment	Sunflower					Pigeonpea					Greengram				
	FYM (Mg ha^−1^)	Mineral fertilizers (kg ha^−1^)			Rock phosphate (kg ha^−1^)	FYM (Mg ha^−1^)	Mineral fertilizers (kg ha^−1^)			Rock phosphate (kg ha^−1^)	FYM (Mg ha^−1^)	Mineral fertilizers (kg ha^−1^)			Rock phosphate (kg ha^−1^)
		N	P_2_O_5_	K_2_O			N	P_2_O_5_	K_2_O			N	P_2_O_5_	K_2_O	
Organic	13.3	0	0	0	0	4.4	0	0	0	167	4.4	0	0	0	167
Integrated	3.3	45	60	30	0	1.1	15	50	0	0	1.1	15	50	0	0
Control*	0	60	60	30	0	0	20	50	0	0	0	20	50	0	0

*Use of chemical inputs alone.

**Table 3:** Amount of nutrients applied each year through farmyard manure and rock phosphate in different crops under organic management.

Crop	FYM (Mg ha^−1^)	Rock phosphate (kg ha^−1^)	Nutrients applied through FYM (kg ha^−1^)			P_2_O_5_ applied through rock phosphate (kg ha^−1^)	Total major nutrients applied (kg ha^−1^)			Total micronutrients applied (kg ha^−1^)			
			N	P_2_O_5_	K_2_O		N	P_2_O_5_	K_2_O	Fe	Cu	Mn	Zn
Sunflower	12.0	0	60	68.7	57.6	0	60	68.7	57.6	5.42	0.33	2.75	1.71
Pigeonpea	4.0	165	20	22.9	19.2	27.2	15	50.1	19.2	1.81	0.11	0.91	0.57
Greengram	4.0	165	20	22.9	19.2	27.2	20	50.1	19.2	1.81	0.11	0.91	0.57

Every year, the crops were sown after the receipt of monsoon rainfall in the month of June. The organic plots were maintained chemical free throughout the years. Manual weeding and manually operated wheel-hoe were used to keep the plots wee free. The prophylactic measure used are described by the authors in their earlier paper (3). Crops were harvested and the yields were converted to pigeonpea equivalent yield (PEY) as per De Wit (27):

$PEY\;(kg\;ha^{-1})$ = [Yield of sunflower or greengram (kg ha^−1^) × price of sunflower or greengram seed (Rs kg^−1^)/price of pigeonpea (Rs kg^−1^)].

### Soil sampling and analysis

A core sampler was used to collect the soil samples before the application of various nutrients and after the crop harvest in 2021 from various soil depts (0–20, 20–40, and 40–60 cm). The soil samples for various microbial activity were kept at 4°C and analyzed within 2 weeks. Bulk density of the soil was determined using metallic cores of known volume. The detailed procedures are explained by the author in their earlier paper (3).

Keen Rackzowski box method was used to determine the WHC and total porosity (28). Potentiometric method as described by Jackson (29) was used for determining soil pH. Available N, P, and K were determined using Kjeldahl method, Olsen’s method and flame photometer method (29–31). Micronutrients were determined using the atomic absorption spectrometry (32). Soil organic carbon was multiplied with BD to arrive at soil carbon stock (33).

Various microbial activities like dehydrogenase (DHA), soil microbial biomass carbon (MBC), urease and acid phosphatase was analyzed/measured using methods of Casida et al. (34), Vance et al. (35), Tabatabai and Bremner (36), and Tabatabai and Bremner (37).

### Crop quality analysis

The dried seeds were stored at room temperature prior to analysis for various parameters. The samples of greengram and pigeonpea were analyzed for chemical parameters after tri-acid digestion. Nitrogen content was determined by Kjeldahl method (38). Protein content was determined by using the formula: N × 5.4 (39). Phosphorus content was analyzed photometrically (40). Potassium content was determined by using flame photometer and micronutrients (Fe, Cu, Mn, and Zn) by using atomic absorption spectroscopy. Sunflower oil was extracted using hexane on Soxhlet apparatus using the methodology of Anjani and Yadav (41).

### Statistical analysis

Using the International Rice Research Institute (IRRI) Star and ANOVA, data were statistically evaluated. Tukey’s HSD *post hoc* comparisons were used to clarify significant differences in means (*p* < 0.05).

## Results

### Crop quality

Different production systems significantly influenced the protein content of pigeonpea seed (Table 4). Pigeonpea grown under integrated production system being on par with organic system recorded significantly higher protein content (20.0%) than that of Control (chemical inputs). However, different production systems had no significant influence on protein content of greengram seed. Organic production system being on par with integrated production system recorded significantly greater P content in both pigeonpea and greengram seeds compared to Control (chemical inputs). Significantly greater K content of both pigeonpea and greengram was recorded with organic production system than other treatments. The micronutrient (Fe, Zn, Cu, and Mn) contents of both pigeonpea and greengram seeds varied significantly with different production systems (Table 4). Organic production system being on par with integrated production system registered significantly higher Fe and Zn contents in seeds of both crops compared to Control (chemical inputs). The Zn and Cu contents of organically grown pigeonpea and greengram seed was greater than that of other production systems.

**Table 4:** Seed protein, P, K and micronutrient contents mas influenced by different production systems.

Production system	Protein (%)	P (%)	K (%)	Fe (ppm)	Zn (ppm)	Cu (ppm)	Mn (ppm)
Pigeonpea							
Organic	19.6 ± 0.1a	0.19 ± 0.02a	2.39 ± 0.01a	35.6 ± 1.5a	25.0 ± 0.2.1a	10.4 ± 1.8a	11.3 ± 2.3a
Integrated	20.0 ± 0.2a	0.18 ± 0.01ab	2.33 ± 0.03a	31.7 ± 2.1ab	22.7 ± 3.1b	9.7 ± 2.2b	10.8 ± 2.0ab
Control	17.9 ± 0.1b	0.17 ± 0.03b	2.20 ± 0.03b	36.7 ± 1.4b	21.8 ± 2.8b	7.9 ± 1.2c	10.2 ± 2.1b
Greengram							
Organic	21.8 ± 0.2a	0.19 ± 0.02a	2.85 ± 0.01a	37.0 ± 1.2a	29.6 ± 2.1a	10.8 ± 1.2a	12.7 ± 2.1a
Integrated	21.4 ± 0.1a	0.19 ± 0.01a	2.61 ± 0.02b	35.4 ± 1.8ab	29.1 ± 1.7b	8.0 ± 1.8b	12.2 ± 1.8ab
Control*	21.2 ± 0.1a	0.17 ± 0.02b	2.59 ± 0.01b	30.9 ± 1.6b	28.8 ± 1.8b	7.2 ± 0.9c	10.7 ± 1.4b

*Use of chemical inputs alone. Means in the same columns with different letters are significantly (*p* < 0.05) different.

**Figure 3:**
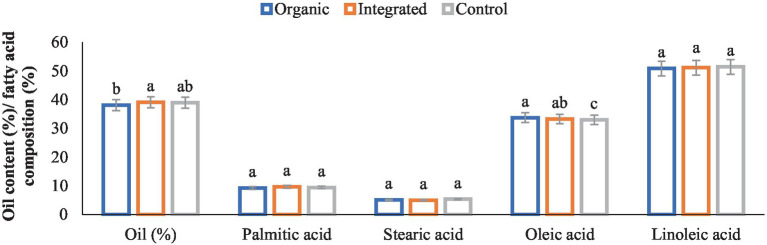
Effect of different production systems on oil content and fatty acid composition of sunflower seed. Bars with different letters within each parameter are significantly (*p* < 0.05) different.

Different production systems had significant effect on sunflower oil content (Figure 3). Integrated production system being on par with Control (chemical inputs) had greater oil content than that of organic production system. Regarding fatty acid composition of oil, organically produced sunflower oil being on par with that of integrated production system had a higher content of oleic acid than Control (chemical inputs). However, no statistical differences were evident among different production systems in terms of palmitic acid, stearic acid and linoleic acid contents.

### Crop yield

**Figure 4:**
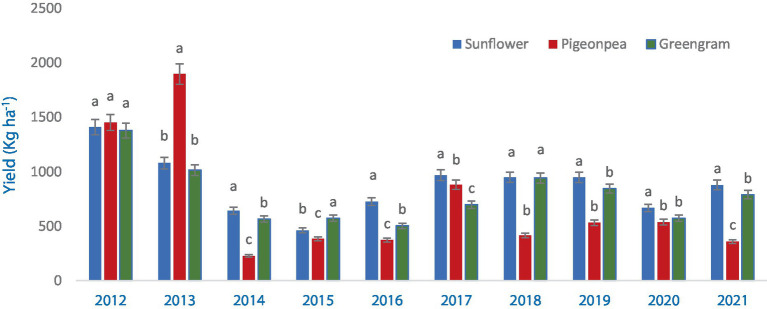
Pigeonpea equivalent yield (PEY) of crops during 2012–2021 (averaged across three production systems). Bars with different letters within each year are significantly (*p* < 0.05) different.

Yield of all the different crops and cropping system was significantly different in terms of pigeonpea equivalent yield (PEY) in all the years (Figure 4). Rainfall distribution and amount has a greater impact on the yield. Sufficient and well distributed rainfall during 2012 and 2013 resulted in higher yield due to less crop stress. Intermittent dry spells and less rainfall during the others years resulted in PEY less than 1,000 kg ha^−1^ (Figure 1).

**Figure 5:**
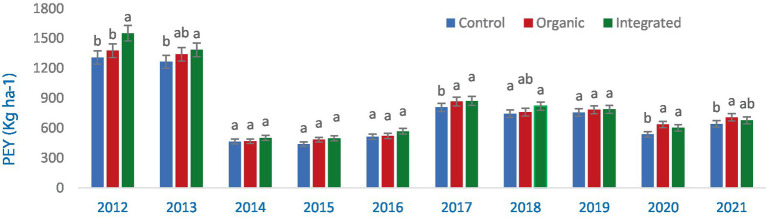
Effect of different production systems on pigeonpea equivalent yield (PEY) of crops during 2012–2021 (averaged across three crops). Bars with different letters within each year are significantly (*p* < 0.05) different.

In the first year, the integrated production system greatly outperformed the organic and control (chemical inputs) treatments in terms of PEY production (2012). In the second year, both integrated and organic production systems were comparable (Figure 5) implying the narrowing of gap. However, no significant changes in PEY was observed during the 2014–2017, presumably as a result of extremely low yields in all treatment groups. When compared to the control (chemical inputs), integrated production system produced more PEY in 2017 and 2018 than an organic production system did (Figure 5). During 2019–2021, both organic and integrated production systems recorded similar but significantly higher PEY than Control (chemical inputs).

### Soil parameters

#### Physical properties

Among the crops, plots under pigeonpea had considerably lower bulk density compared to other crops after 10 years. Plots under organic production system and integrated production system had significantly lower bulk density of soil than that under control (chemical inputs) (Table 5). Cultivation of pigeonpea crop resulted in significantly higher soil porosity (54.40%) compared to other crops. Plots managed organically had significantly higher porosity (53.79%) than control (chemical inputs) across the production systems. The soil’s ability to retain water was also noticeably higher in the plots grown with pigeonpea crops. When comparing the various production systems, organically managed soils had much more water retention capacity than the control (chemical inputs) (Table 5).

**Table 5:** Effect of crops and production systems on soil physical parameters.

Treatment	Bulk density (Mg m^−3^)	Porosity (%)	Water holding capacity (%)
Crop			
Sunflower	1.25 ± 0.1a	51.31 ± 0.2b	37.13 ± 0.2b
Pigeonpea	1.20 ± 0.2b	54.40 ± 0.1a	38.94 ± 0.2a
Greengram	1.21 ± 0.1ab	51.09 ± 0.3b	37.45 ± 0.2b
Production system			
Control*	1.26 ± 0.0a	51.00 ± 0.1b	37.21 ± 0.1b
Organic	1.18 ± 0.2b	53.79 ± 0.2a	38.72 ± 0.2a
Integrated	1.21 ± 0.2b	52.41 ± 0.1a	37.84 ± 0.1b

*Use of chemical inputs alone; initial bulk density was 1.21 Mg m^−3^; means in the same columns with different letters are significantly (*p* < 0.05) different.

#### Chemical properties

The 10-year long experiment had no significant effect on soil pH, although pH was marginally higher in the plots under organic management (Table 6). The soil organic C (SOC) content in the plots under organic production system was 32.6% more than the initial organic carbon of the soil, with higher soil N. The SOC was significantly higher in FYM amended plots compared with mineral fertilizer and integrated production treatments. Plots under integrated production system had higher soil P compared with other treatments (Table 6). However, plots under organic production system being on par with integrated system had significantly higher K content than under control (chemical inputs) plots (Table 6). In our study, DTPA-extractable micronutrient (Cu, Mn, Fe, and Zn) contents were significantly higher in the plots under organic production system than under other treatments.

**Table 6:** Effect of different production systems on soil properties.

Production system	pH	Organic C (%)	Av. macronutrients (kg ha^−1^)			DTPA-extractable micronutrients (ppm)				DHA (μg TPF g^−1^ soil h^−1^)	MBC (μg g^−1^ soil)
			N	P	K	Cu	Mn	Fe	Zn		
Control*	6.45 ± 0.15a	0.42 ± 0.02c	188.1 ± 38c	24.3 ± 22b	217.2 ± 8b	1.64 ± 0.2b	19.0 ± 0.2c	6.8 ± 0.6c	0.43 ± 0.2c	3.61 ± 0.2c	252.8 ± 38b
Organic	6.63 ± 0.18a	0.57 ± 0.03a	205.2 ± 16ab	25.2 ± 11a	244.8 ± 12a	2.08 ± 0.7a	29.9 ± 0.6a	13.8 ± 0.1a	0.68 ± 0.4a	5.86 ± 0.6a	317.3 ± 26a
Integrated	6.55 ± 0.14a	0.49 ± 0.03b	191.5 ± 25bc	26.5 ± 23a	231.4 ± 16a	1.83 ± 0.3a	26.7 ± 0.4b	11.5 ± 0.4b	0.55 ± 0.1b	4.99 ± 0.2b	298.5 ± 31a
Initial values	6.51	0.43	179.0	24.7	218.1	1.69	20.5	6.6	0.47	3.56	262.4

*Use of chemical inputs alone; means in the same columns with different letters are significantly (*p* < 0.05) different.

#### Biological properties

**Figure 6:**
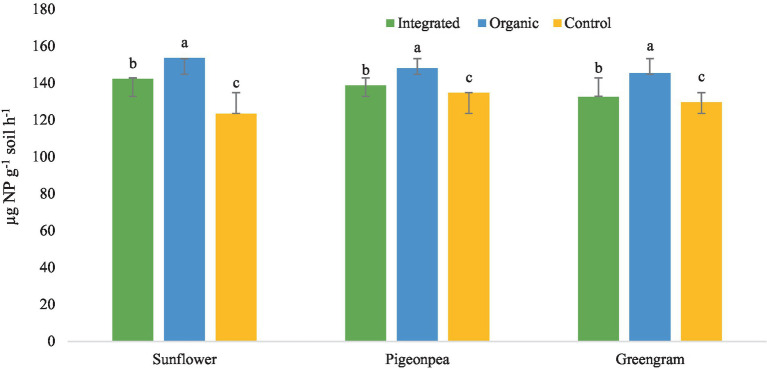
Effect of different production systems and crops on soil acid phosphatase activity. Bars with different letters within each crop are significantly (*p* < 0.05) different.

**Figure 7:**
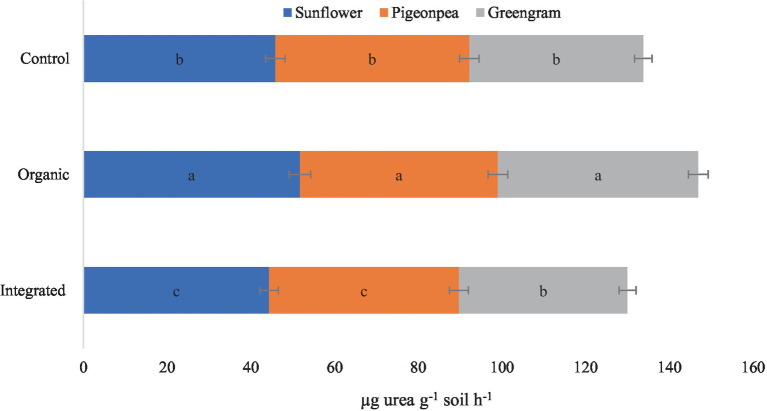
Effect of different production systems and crops on soil urease activity. Bars with different letters within each crop are significantly (*p* < 0.05) different.

Under this experiment, we observed higher DHA and MBC in plots under organic production system than under other systems for all the three crops (Table 6). Improved DHA, in our study, in plots under organic production system is a result of diversified nutritional amendments which led to the improvement of soil biological health. Similarly, higher activity of acid phosphatase with organic production system (Figure 6) might be attributed to the accelerated microbial activity due to improved organic carbon content of the soil. However, increased level of urease enzyme under organic management (Figure 7) suggested persistent availability of substrates with C-N bonds for the enzyme to work.

## Discussion

### Crop yield and quality

**Figure 8:**
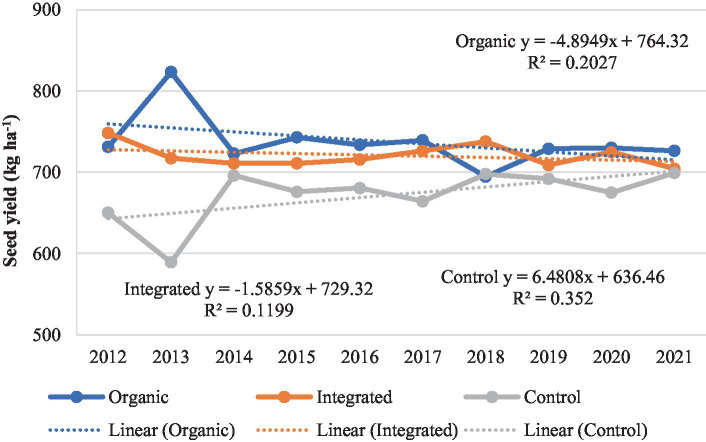
Performance of pigeonpea under different production systems during 2012–2021.

**Figure 9:**
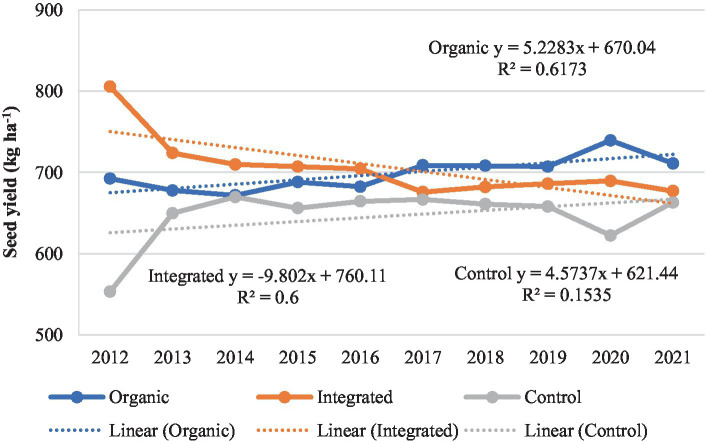
Performance of greengram under different production systems during 2012–2021.

In our study, the performance of crops varied under different production systems. When the yield data was adjusted for year effect, pigeonpea performed better under organic production system than under other treatments across all the years except 2018 (Figure 8). Averaged across the years, pigeonpea seed yield (721–737 kg ha^−1^) was similar under both organic and integrated production systems compared to Control (chemical inputs) (672 kg ha^−1^). In greengram, the seed yields were higher under integrated production system during initial 5 years, whereas organic system recorded marginally higher yields during the latter 5 years compared to other treatments (Figure 9). On average, greengram seed yield (699–706 kg ha^−1^) was similar under both organic and integrated production systems. Results here are compared with past studies (3, 42–45), where organic crop yields were lower than conventional crop yields during initial years. As the nutrient cycling processes in organic systems change from inorganic N fertilization to organic amendments, lower crop yields in the plots under organic production systems may have been related to the less readily available nutrients in the early years of transition (46–49).

**Figure 10:**
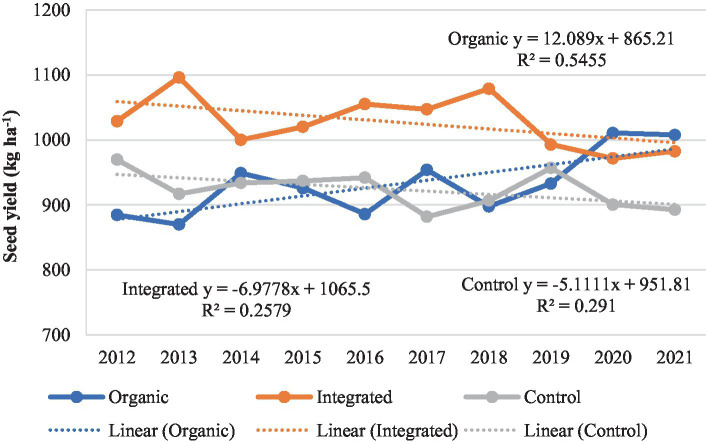
Performance of sunflower under different production systems during 2012–2021.

However, integrated production system recorded higher seed yield of sunflower in all the years except during last 2 years where seed yield was marginally higher under organic production system than other treatments (Figure 10). A gradual improvement in seed yield of sunflower was observed under organic production system whereas, the seed yield showed a declining trend in the plots under Control (chemical inputs), over the years. The yield gap between organic and integrated production systems narrowed down after 8 years of study. Integrated production system, averaged across the years, recorded 9.2–10.0% higher seed yield than that of organic and Control (chemical inputs) treatments. Many comparisons between organic and conventional production systems are mostly from relatively short-term experiments (50, 51). However, there are few well documented long-term (more than 10 years) comparisons between organic and conventional production systems. Similarly to our study, Schrama et al. (45) reported that the yield gap between organic and conventional production systems declined during a 13-year experimental period, suggesting that the yield gap between organic and conventional production systems may decline over time.

A large number of studies have been reported that attempt to investigate if there is a difference in the nutritional value of organically and conventionally grown food (52, 53). In general, our results showed marginally lower protein, and higher K and micronutrient (Fe, Zn, Cu, and Mn) contents of both pigeonpea and greengram seeds under organic management than other treatments. Gopinath et al. (54) and Saha et al. (55) also reported similar results. According to Worthington (56), organic produce from various crops had 21% more iron, 29% more magnesium, and 13.6% more phosphorus. According to a study by Lairon (53), organic food has 21 and 29% more iron and magnesium than non-organic food. In sunflower, integrated production system being on par with Control (chemical inputs) had greater oil content than that of organic production system. However, different production systems had no significant effect on fatty acid composition except that organic sunflower oil had higher content of oleic acid.

### Soil properties

Numerous desirable soil characteristics, such as a reduction in bulk density, increased porosity, and increased water-holding capacity, have been linked to the use of organic amendments (14, 16, 26, 45, 57, 58). According to earlier studies (16, 18, 19, 59), organic production systems had higher pH levels in mildly acidic soils than their conventional counterparts. Our findings on the impact of organic systems on various chemical properties of soil are similar to those of those earlier studies.

One of the important environmental benefits due to a shift from conventional to organic production systems is an improvement in soil carbon content (11, 60). The SOC was significantly higher in the plots under organic management compared with mineral fertilizer and integrated production treatments. This increment in SOC might be attributed to the direct addition of organic source of plant nutrients which in turn led to lesser mineralization owing to its wider C: N ratio (61, 62). Previously, some long-term experiments reported notable improvements in SOC content through incorporation of organic manures (63). In a similar line, Aoyama et al. (64) observed increased level of organic matter in soil after 18 years of experimentation with the addition of organic manure. Hati et al. (65), Ramesh et al. (66), and Gopinath et al. (3) also reported higher SOC with organic nutrients application on long term basis.

Higher soil P was found in integrated system as reported by Chen et al. (67). Slower release of organic materials, particularly during initial years under organic production results in lower availability of plant nutrients in organic plots (47, 48). Patel et al. (68) also reported an increase in available P with integrated application of NPK and FYM in a long-term experiment with soybean-wheat cropping system. On the other hand, available K was found higher in organic system. Greater available K with organic nutrition has been documented by Bulluck et al. (57) and Panwar et al. (69). This beneficial effect with organic manure application might be attributed to organic source induced release of organic colloids with more cation exchange sites which adds up more amount of available K by attracting them from the non-exchangeable pool (70). The improved agricultural practices such as soil organic amendments play vital role in soil micronutrient availability (22). Higher DTPA-extractable micronutrient (Cu, Mn, Fe, and Zn) contents under organic production system may be attributed to FYM addition and enhanced soil microbial properties might have improved the micronutrient status of the soil.

Soil organic carbon (SOC) is consisted of a vital fraction termed as microbial biomass carbon (MBC) of soil (71). Microbial biomass carbon (MBC) and dehydrogenase activity (DHA) are crucial indicators for soil quality (72). These indicators also provide clear reflection of soil microbial activity, specifically representing the metabolically active fraction of soil microbial population (73, 74). Improved MBC might be attributed to the property of organic manure to be a more soluble source of substrate for better microbial proliferation in soil (75). Accordingly, implementation of organic management might have accelerated the availability of substrates and stimulated the metabolic activity of soil microbes, which leads to enhanced dehydrogenase activity as confirmed by the outcomes of Basak et al. (76). The reason behind this stimulation of soil dehydrogenase may be due to addition of substrates containing several intra- and extra-cellular enzymes through the incorporation of organic manures. Similar result was also reported by Saviozzi et al. (77) and Smitha et al. (78). Similarly, phosphatase activity in soil is likely to get amplified in response to organic nutrient management compared to chemical inputs (25, 79). In our study, higher activity of acid phosphatase with organic production system might be attributed to the accelerated microbial activity due to improved organic carbon content of the soil. However, increased level of urease enzyme under organic management may be because of persistent availability of substrates with C-N bonds for the enzyme to work. Few other researchers have also reported similar type of improvement in urease activity with the application of organic manures (80–82).

## Conclusion

Long-term research based recommendations must be developed for suitable production system that provide higher crop yields, seed quality and improve soil fertility in rainfed areas of India’s semiarid tropics. The results of 10-year experiment revealed that the crop yields were lower under organic production system than that of other production systems, particularly during initial years. The yield gap of both the legumes (pigeonpea and greengram) between organic and integrated production systems was less even during initial years, indicating that they may be better suited for organic production under rainfed areas. In general, yield gap of all the three crops between organic and conventional production systems declined over the years. Organic production also improved most of the quality parameters of pigeonpea, greengram, and sunflower relative to integrated production system. Consumption of organic produce, therefore, is one of the approaches to address nutritional security particularly the micronutrient malnutrition of the people. Organic management improved soil properties such as bulk density, porosity, water holding capacity, organic carbon, micronutrient contents and soil microbial activities. In general, the soil fertility parameters were the poorest under Control (use of chemical inputs alone). We conclude that, in the long-run, organic farming has the potential to improve crop yields and soil properties in rainfed semiarid tropics of India.

## Data availability statement

The original contributions presented in the study are included in the article/supplementary material, further inquiries can be directed to the corresponding authors.

## Author contributions

KG: conceptualization. KG and GV: methodology. MM, MJ, KG, and BMR: formal analysis. KG, GV, and TP: investigation. MP: resources. KG and VK: data curation, writing, review, and editing. GC: supervision. VS: project administration. All authors have read and agreed to the published version of the manuscript.

## Funding

This work was financially supported by grants from the Indian Council of Agricultural Research (ICAR), New Delhi in the form of the National Innovations in Climate Resilient Agriculture (NICRA) Project (Grant No. 2–2(201)/17–18/NICRA).

## Conflict of interest

The authors declare that the research was conducted in the absence of any commercial or financial relationships that could be construed as a potential conflict of interest.

## Publisher’s note

All claims expressed in this article are solely those of the authors and do not necessarily represent those of their affiliated organizations, or those of the publisher, the editors and the reviewers. Any product that may be evaluated in this article, or claim that may be made by its manufacturer, is not guaranteed or endorsed by the publisher.
